# Innovation through networking: the single contact points for employers in the German vocational rehabilitation and participation system

**DOI:** 10.3389/fresc.2025.1659687

**Published:** 2025-12-12

**Authors:** Jana York, Sarah Lamb, Sarah Schulze, Jan Jochmaring, Jörg-Tobias Kuhn, Bastian Pelka

**Affiliations:** 1 Research Unit Sociology of Rehabilitation and Participation, Department of Rehabilitation Sciences, TU Dortmund University, Dortmund, Germany; 2 Research Unit Methods of Empirical Educational Research, Department of Rehabilitation Sciences, TU Dortmund University, Dortmund, Germany

**Keywords:** single contact points for employers, system of vocational rehabilitation and participation, social innovation, employer counseling, mixed methods

## Abstract

**Introduction:**

The German vocational rehabilitation and participation system is highly complex due to fragmented legal responsibilities and institutional structures. To address this and improve labor market inclusion for people with severe disabilities, Single Contact Points for Employers [Einheitliche Ansprechstellen für Arbeitgeber (EAA)] were established in 2022. The EAA are legally mandated, provider-independent entities that support employers in hiring and retaining employees with disabilities. The article analyzes the network structure of the EAA within the vocational rehabilitation and participation system, how the structure is evaluated, and which partners are most relevant to the EAA.

**Methods:**

A network analysis was conducted through egocentric network maps (*N* = 20) with EAA consultants, online surveys of EAA consultants (*N* = 18), and their key network partners (*N* = 123). Additionally, *N* = 7 guided expert interviews (5 with employers, 2 with EAA supervisors) and three focus groups of 3–6 people each (1 with EAA consultants, 1 with EAA supervisors, and 1 with network partners) were analyzed using qualitative content analysis to assess the structure and quality of cooperation.

**Results:**

The EAA work closely with policy actors from the vocational rehabilitation and participation system who provide advice and services for participation in work, such as Integration Services, Employment Agencies, and Specialist Agencies. These collaborations are characterized by mutual information exchange and generally positive cooperation. Actors from the economy, civil society, and academic sector are underrepresented in the EAA's overall network. Employers’ associations were rarely named as key partners, and civil society organizations were absent.

**Discussion:**

The EAA's strong integration into policy networks underscores their institutional relevance. However, their limited ties to economic and civil entities suggest a constrained capacity for innovation. This pattern reflects both the strengths of their legal mandate and the challenges of acting independently within a highly structured system. Expanding their relational scope could foster more holistic solutions for inclusive employment.

## 1 Introduction

The German vocational rehabilitation and participation system is highly complex, reflecting its fragmentation across legal entitlements, institutional responsibilities and diverse support services (1–4). Stakeholders often refer to it as a “support jungle” (5). System complexity and lack of knowledge regarding advice, information and support services are among the reasons why people with disabilities^1^ still have fewer opportunities to participate in work (6–9).

Recent findings on the employment situation of people with (severe) disabilities indicate that the German vocational rehabilitation and participation system falls short of fulfilling its legal obligation to ensure an inclusive labor market (10). In 2021, the employment rate of severely disabled people was 49.8%, which is lower than the employment rate of the overall population, which was 78.7% at the same time (6). The unemployment rate for severely disabled people is also higher than the German average (11), despite the fact that unemployed people with severe disabilities are, on average, better qualified than those without disabilities (6).

To address the complexity of the system and increase opportunities for people with severe disabilities to participate in work, a new instrument was introduced in the vocational rehabilitation and participation system. In 2022, the Single Contact Points for Employers (EAA) were implemented by law with Section 185a of the Ninth Social Code [Neuntes Sozialgesetzbuch (SGB IX)]. The EAA are intended to serve as support services for current and prospective employers of individuals with severe disabilities. They “inform, advise, and support employers in the training, recruitment, and employment of severely disabled people” (Section 185a SGB IX). They have the following tasks:

1. “To address employers and sensitize them to the training, recruitment and employment of severely disabled people,
2. To be available to employers as an independent guide on issues relating to training, recruitment, career support and securing employment for severely disabled people, and
3. To support employers in submitting applications to the responsible service providers” [Section 185a (2) SGB IX].

The EAA are financed as accompanying assistance in working life by means of the equalization levy [Section 185a (2) SGB IX]. Employers who fail to meet their employment obligations or do not sufficiently employ severely disabled people must pay this levy.

The already complex vocational rehabilitation and participation system has become even more intricate and complex with the implementation of the EAA. However, the EAA aim to address this complexity more effectively by serving as provider-independent guides. Provider-independent guidance means the EAA do not act as service or cost providers within the vocational rehabilitation and participation system, but they are still required to understand the support structures of all relevant stakeholders (5) and actively network with them. In their practical work, the EAA adopt a so-called “pilot's perspective”, which enables them to identify alternative paths alongside the “well-trodden paths” of the system and to identify and take shortcuts to support employees with disabilities. This piloting approach represents a reconfiguration of social practices aimed at finding new pathways and improved solutions, positioning the EAA as a form of social innovation (12–14).

“A social innovation is a new combination and/or new configuration of social practices in certain areas of action or social contexts prompted by certain actors or constellations of actors in an intentional targeted manner with the goal of better satisfying or answering needs and problems than is possible on the basis of established practices” (13).

From an innovation theory perspective, questions arise about the EAA's potential to drive innovation and their capacity to act as “innovators” (15), opening new pathways to labor market inclusion. At the same time, from an innovation theory perspective, the complex, sometimes long-established constellations of actors in the so-called innovation ecosystem (16–18) of vocational rehabilitation and participation are of interest.

“An innovation ecosystem is the evolving set of actors, activities, and artifacts, and the institutions and relations, including complementary and substitute relations, that are important for the innovative performance of an actor or a population of actors” (18).

The specific interaction among different actors within an ecosystem can either foster or hinder innovation (19). Innovation ecosystems can be analyzed using helix models. Although various helix models (16, 17, 20, 21) differ in the type of actors they include, they share a core characteristic: Multipolarity, which forms the framework for the emergence of innovations (5). This study applies Carayannis and Campbell's (16) quadruple helix model to analyze the vocational rehabilitation and participation ecosystem, which differentiates actors in the sectors of politics, economy, civil society, and academia. Carayannis and Campbell (16) formulate the hypothesis that innovations are more successful when actors from all four helix sectors are involved in the innovation process. Hence, the current study investigates how the EAA, as a new social practice, is linked to different actors.

The EAA belong to the policy sector, as they are legally mandated and publicly funded through the equalization levy (5). In accordance with their legal mandate, the EAA are supposed to guide employers through the complex ecosystem of vocational rehabilitation and participation. In order to “navigate” the system and explore “new paths”, the EAA not only require knowledge about the funding and support services of all funding bodies and service providers in the system, but also personal network contacts in order to initiate and moderate application procedures. This research perspective is grounded in the assumption that innovation within the vocational rehabilitation and participation ecosystem depends on effective networking.

This article focuses on the EAA's networking with the key actors of the vocational rehabilitation and participation ecosystem in the region of the Rhineland Regional Association in North Rhine-Westphalia. This is based on data from the project “Evaluation of the Single Contact Points for Employers, viewed as a social innovation in the ecosystem of the rehabilitation system and labor market [Evaluation der Einheitlichen Ansprechstellen für Arbeitgeber*innen, betrachtet als soziale Innovation im Eco-System von Rehabilitationssystem und Arbeitsmarkt (EvaEfA)]”, funded by the Inclusion Office of the Rhineland Regional Association [Landschaftsverband Rheinland Inklusionsamt (LVR Inclusion Office)].

The EvaEfA project examines both the predecessor structures of the EAA^2^ and their cooperation with other key actors in the ecosystem.

The following research questions are of interest in this article:

1. How intensively are the EAA's interlinked within the network of the vocational rehabilitation and participation ecosystem? How are networking activities of relevant actors assessed?
2. Who are the EAA's most important network partners in the vocational rehabilitation and participation ecosystem? Why are these network partners particularly important for the EAA?
3. How is cooperation, communication and the exchange of information between the EAA and its most important network partners in the vocational rehabilitation and participation ecosystem structured?

## 2 Method

The EvaEfA project uses a participatory dialogic procedure (5) to investigate the function of EAA within the complex vocational rehabilitation and participation ecosystem. The participatory dialogic procedure solves two challenges in researching EAA within the complex vocational rehabilitation and participation system. First, it stimulates discussion about the key actors in the system. Second, it makes implicit knowledge that exists in the field, which is partly embedded in routines or experiential knowledge and therefore difficult to grasp using traditional empirical approaches, explicit. The participatory dialogic procedure is based on participatory research approaches (22), co-creation (23), transformative research (24) and dialogic methods (25). The aim of the procedure is to enrich the research results with the practical experience of relevant experts and a communicative validation (26, 27) of central research results. To achieve this, the participatory dialogical process incorporated recurring dialog formats such as workshops, focus groups or monthly meetings (28).

To examine the function of the EAA within the vocational rehabilitation and participation ecosystem, the study uses a mixed-methods design combining qualitative and quantitative data (29). The goal of integrating different types of data is to develop a comprehensive understanding of the EAA, capturing both the depth and breadth of their implementation. The following section presents two components of the mixed-methods design in more detail: Network analysis and the expert interviews. Other results of the research project can be found in the articles mentioned here. These include the historical development of the EAA (see text footnote 2), networking among individual EAA consultants (30–32), the response of the vocational rehabilitation and participation ecosystem to the EAA (5), and the participatory dialogue procedure of the research project (5).

### 2.1 Network analysis

#### 2.1.1 Data collection

The EvaEfA project examines the EAA's overall network in the Rhineland. An overall network analysis is characterized by the fact that the nodes (actors in the network) and their edges (connections between the actors) are explicated within predefined network boundaries (33). Relationships to other nodes outside the defined boundary are not considered. Network boundaries must be clearly justified (33), typically based on organizational or group membership criteria (34). As it was not possible to determine the EAA network in the Rhineland *a priori*, data collection followed three key steps:

1. Survey of egocentric network maps of all EAA consultants (30–32),
2. Online survey of EAA consultants,
3. Online survey of the most important EAA network partners.

The three steps are explained below.

1. Survey of egocentric network maps of all EAA consultants

The EAA consultants in the Rhineland form the entry point for data collection. EAA consultants were personally approached by project staff during information workshops, which form part of the participatory dialogue process, and were invited to participate in the study. During another workshop (30), EAA consultants (*N* = 20) developed egocentric network maps, identifying actors that play a role to their everyday professional activities (30–32). Based on the 20 network maps, a list comprising 700 network contacts (including duplicates) across 32 institutions or categories was compiled (31). This formed the basis for the name list used in the next steps of the analysis. This list served to define the boundaries of the overall network. For this list of names and actors, network partners were selected that were mentioned particularly frequently by the EAA consultants, e.g., the Integration Services [Integrationsfachdienste (IFD)], Specialist Agencies for People with Disabilities in Working Life (Fachstellen Behinderte Menschen im Beruf), Action Inclusion by the Inclusion Office (Aktion Inklusion des Inklusionsamtes), and the Employment Agencies [Agenturen für Arbeit (AA)]. All of the institutions mentioned as examples here have a political mandate and aim to increase workplace participation among people with disabilities. In addition, institutions and network partners with which the EAA consultants are employed were taken into account: the Chamber of Skilled Crafts [Handwerkskammer (HWK)], the Chamber of Industry and Commerce [Industrie- und Handelskammer (IHK)], the Chamber of Agriculture [Landwirtschaftskammer (LWK)], and the Advanced Training Academy for Business [Fortbildungsakademie der Wirtschaft (FAW)]. As public-law corporations, chambers are professional organizations that perform self-governing tasks within specific professional groups or industries. In addition, institutions that were mentioned by the EAA consultants in the network maps and which were considered to be of central importance for the EAA from an innovation theory perspective in the participatory dialogue process, such as employers’ and entrepreneurs’ associations, were also taken into account. The total list of names included 14 different actors. The Employment Agencies and the Inclusion Office are divided into departments that are important for the EAA. For this reason, four additional subdivisions were listed within the Employment Agencies. The same was done for the Inclusion Office.

1. Online survey of EAA consultants

In a second step, 18 of the 20 EAA consultants who had previously worked out a network map once again selected their key network partners from this list of names or actors in an online survey. They answered seven questions regarding cooperation, communication and information exchange with these selected partners. This selection of core partners and the consultants’ assessments of collaboration formed the basis for analyzing the overall network.

1. Online survey of the most important EAA network partners

In the third step, EAA's network partners (*N* = 123) completed an online questionnaire. EAA consultants assisted in distributing the survey to ensure it reached the appropriate network partners. The survey was forwarded by the EAA consultants directly to their key network partners. In turn, the EAA's network partners were asked to indicate which EAA consultants they are in contact with and to select key network partners from the list described above. The surveyed institutions also answered seven questions in order to be able to map the cooperation, communication and exchange of information between the EAA consultants and the network partners from both perspectives (see Table 1).

**Table 1:** Excerpt from questionnaire.

How would you describe the communications between you and [network partner/EAA]?			
○ formal	○	○	○ informal
How would you rate the efficiency of the collaboration with [network partner/EAA] in terms of achieving common professional goals?			
○ inefficient	○	○	○ efficient
How quickly do you receive the information from [network partner/EAA]?			
○ very slow	○ rather slow	○ rather fast	○ very fast

[Network partner/EAA] = Placeholder. Questions were presented in a system-generated manner depending on the actors selected from the list of names.

#### 2.1.2 Data analysis

The evaluation and visualization of the overall network were based on combined data from the online surveys of EAA consultants and their network partners. The statistical software R (R Core Team, 2025) was used for data preparation and analysis. A web-based tool was used to visualize the network structures (35, 36).

The positioning of the nodes in the network is based on the Fruchterman-Reingold procedure (37). This procedure ensures that connected nodes are positioned close to each other and unconnected nodes are positioned further away. In order to obtain a well-structured and readable network map, the positioning is also iterative, whereby the nodes are positioned and moved on the surface until no better visual solution is possible (35). Each node's position depends on the positions of all other nodes in the network. The following therefore applies to the visualization: There is no defined starting point, the distances between the nodes are not linear, and the aim is to reveal latent network structures. In addition, the density was calculated, which indicates the ratio of the actually existing edges to the purely theoretically possible edges between the nodes within a network. Density values range from 0 to 1; a value of 0, which means there are no edges between nodes. A value of 1 indicates maximum interconnectedness, meaning all nodes in the network are connected (38). Density represents the proportion of actual connections (edges) relative to all possible connections and is independent of the number of nodes.

### 2.2 Expert interviews

#### 2.2.1 Data collection

To explore how the EAA might foster innovation in work participation, seven interviews were conducted: five with employers and two with EAA supervisors. Three guided focus group interviews were conducted as well: one with EAA consultants, one with EAA supervisors, and one with network partners. Each focus group consisted of three to six people. The interview guidelines addressed various topics. For example, the respondents were asked about the following aspects of the EAA: working methods, benefits, implementation status, cooperation, dissemination and implementation.

##### 2.2.1.1 Recruitment

The EAA consultants were recruited for the focus group using a purposive sampling method as part of the participatory dialogue process. A concerted effort was made to ensure a diverse array of professional backgrounds, geographical locations (urban and rural), and industries. The focus group of supervisors was approached and recruited via the EAA steering group. Two supervisors were unable to participate in the focus group due to scheduling conflicts and were interviewed individually. The focus group of network partners was recruited using contact information that participants could provide voluntarily in the online survey if they were interested in participating in the research project. The employers were interviewed individually and deliberately selected using a documentation tool in which all consulting cases are archived. The size of the company, the industry, and the number of employees with severe disabilities were considered among other factors. A sample of employers was initially approached by EAA consultants to ascertain their interest in participating in the study. In the event that the subjects exhibited interest and granted consent for their contact information to be disseminated, the employers were contacted by project personnel via letter or telephone.

##### 2.2.1.2 Conducting the interviews

The focus groups and interviews were all conducted digitally using video conferencing software. Two project staff members organized the focus groups. Each staff member was responsible for a specific task: one was responsible for technical matters and taking minutes, while the other was responsible for moderating the discussion. The interviews were conducted by one member of the project staff. The administration of both the focus groups and the interviews was facilitated by interview guides. The guidelines addressed various topics. For example, the respondents were asked about the following aspects of the EAA: working methods, benefits, implementation status, cooperation, dissemination and implementation.

##### 2.2.1.3 Documentation

Audio recordings were made of all focus groups and interviews. Subsequently, the audio recordings were transcribed using specialized software.

#### 2.2.2 Data analysis

Interview data were analyzed using a summarizing qualitative content analysis approach (39) which condensed the material into key thematic statements. The analysis yielded a category system representing the core themes expressed by interviewees.

First, the data were segmented into distinct units of analysis. These were determined purely semantically. A unit of analysis was defined here as any text unit that has a thematic context. This could include an entire response or multiple themes within a single answer.

A step-by-step coding process was then applied to interpret the data. To this end, the categories were first formed according to the guidelines and then applied deductively to the material. For data segments that did not align with the deductively derived categories, five additional categories were developed inductively.

The focus group results were summarized into a 13-category system with 18 subcategories. Subcategories differentiate main categories based on theme or characteristics (e.g., positive or negative). Four main categories and subcategories are presented here with their anchor examples, which are particularly relevant for answering the above-mentioned research questions: (1) function of EAA in vocational rehabilitation and participation system, (2) function of EAA in their own institution, (3) cooperation with EAA and (4) network of EAA (see Table 2).

**Table 2:** Relevant categories expert interviews.

No. Main and subcategories	Anchor examples
Function of the EAA in the system of vocational rehabilitation and participation	*“The colleagues and colleagues.. prepare.. the applications for the employers really, as far as I can tell, always really perfectly, so that it can then go through relatively quickly without there being any major queries from the institutions that are supposed to approve something.”*
Function of the EAA in their own institution	*“Well, I would say that the EAA basically work very independently—I think the previous speaker has already said this.”*
2.1 Benefits in the institution	*“In the meantime, of course, we've learned a lot from the joint discussions, but I don't presume to be able to give advice.”*
2.2 Benefits of your own work	*“Ultimately,* we *are the customer at the agency [for employment, note], and the [EAA, note] are the links in between for us. This means that when employers have contacted us and need advice, our colleagues come to us.”*
2.3 Motives of the EAA office at the executing agency	*“In our case, our sponsor went to great lengths to ensure that we were able to get exactly that as an advisory service.”*
2.4 Function of the supervisors	*“And conversely, that also gives me the confidence and trust to let the employee walk on a long leash, and that works brilliantly.”*
Cooperation with EAA	*“Because we often have to deal with jobs as employers, and you always have the feeling that you have to ask two, three, four, five times until something happens, and that wasn't the case in one of the few cases, and it was always an exchange.”*
3.1 Description of the cooperation	*“The EAA then actually listed the funding options, the integration grant, and if something is needed for the person, that you can then also get the appropriate funding. So he explained all these things.”*
3.2 Positive and negative aspects of cooperation	*“But first of all, I think the structure, team and individual advice for employers work very well from my perspective.”*
3.3 Expectations of cooperation with the EAA	*“You want a good collaboration, and it's one-sided, and that was actually my hope, my expectation and my wish.”*
3.4 Termination of the case	*“For me, the case is over when the employee is well provided with everything she needs to really work to the best of her ability and without restrictions, including her personal satisfaction.”*
3.5 Tasks of the EAA	*“The EAA is really only responsible for factual processing.”*
EAA network	*“Because I get requests like that, sometimes from the employment agency, from the rehab advisors, asking if I have any ideas about which other companies could be contacted. I can of course refer them to our training advice service.”*
4.1 Cooperation with the specialist inclusion service	*“With the specialist advisors from the Integration Services, it was the case that I really had to get to know them all and have individual discussions, so they don't necessarily have to pass on all the employer cases to me, but that's up to them.”*
4.2 Cooperation within the network	*“What I find difficult about the role of institutions, the EAA, is that I actually see us, regardless of the provider, as a body for disabled people in working life. The EAA is of course primarily for employers, but in that sense, I see us as one big team.”*

## 3 Results

The results are structured according to the research questions. The findings from the expert interviews are presented here in combination with the results of the network analysis. These results have previously been validated through communication within the participatory dialogic procedure (5).

### 3.1 Network density and quality

1. How intensively are the EAAs interlinked within the network of the vocational rehabilitation and participation ecosystem? How are networking activities of relevant actors assessed?

**Figure 1:**
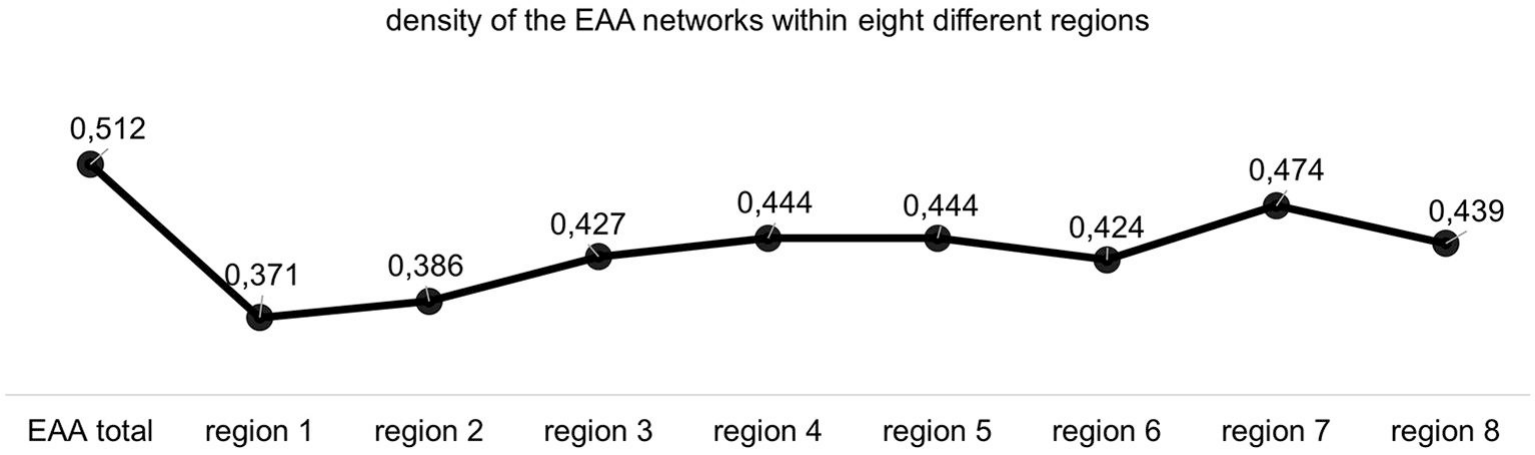
Density of the EAA network within eight different regions.

To address this question, network density was calculated as an indicator of the overall intensity of the network. Figure 1 shows the respective densities of the EAA networks within eight different regions in the Rhineland Regional Association (see Figure 1).

The results show that the network density varies slightly between the regions. Region 7 exhibits the highest density (0.474), indicating that nearly 50% of all possible connections are realized. The lowest density is found in region 1, which shows the lowest density (0.371), meaning fewer than 40% of potential connections are present. The “EAA total” value reflects the aggregated density across all locations, resulting in a higher overall value.

The qualitative analysis of interview data shows that networking and cooperation within the network are rated positively, especially in regions with a longer network history. As one EAA counselor noted in a focus group: “We were involved in a network right from the start, not nationwide, but with a relatively high level of participation from the beginning”. The focus group of network partners highlighted the positive aspects of “cooperation among each other” and “mutually passing on advice requests, even for specialized technical questions”. Sharing is already practiced and supported by “joint data storage” (focus group network partners). In well-established networks, partners are regarded as colleagues with whom counselors work toward a common goal.

### 3.2 Important network partners

1. Who are the EAA's most important network partners in the vocational rehabilitation and participation ecosystem? Why are these network partners particularly important for the EAA?

The egocentric network maps revealed five institutions as particularly important to the EAA's work (31). These include: Integration Services, EAA, Inclusion Office of the Rhineland Regional Association, Specialist Agencies for People with Disabilities in Working Life and Employment Agencies.

**Figure 2:**
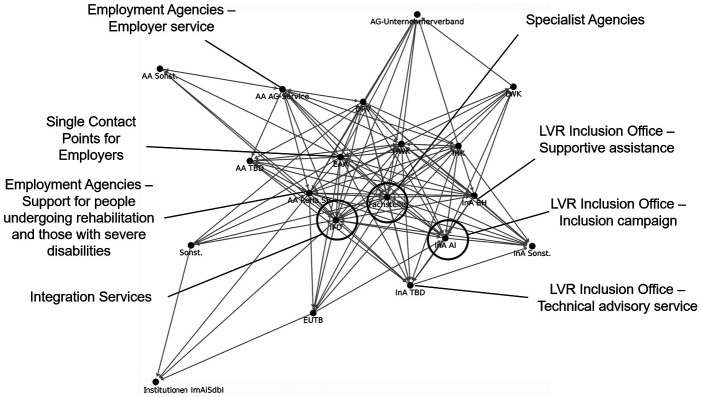
EAA's core network.

All five institutions occupy central positions in the EAA's overall network (see Figure 2).

The Specialist Agencies, Integration Services, and Employment Agencies occupy central positions in the network, indicating that their significance extends beyond their direct collaboration with the EAA. The network visualizations illustrate not only EAA connections but also interconnections among institutions themselves. Thus, these institutions serve as key nodes for multiple stakeholders within the vocational rehabilitation and participation ecosystem. They are important because they complement each other's functions in mediating between employers and individuals with disabilities, thereby enhancing coordination within the ecosystem.

This is supported by focus group interviews with EAA's network partners, including representatives from Employment and Specialist Agencies. From the perspective of the Employment Agencies (support for rehabilitants and severely disabled persons—Reha SB), there are notable distinctions and complementarities between the EAA and Employment Agencies: The Employment Agencies are more responsible for “initiating employment”, whereas the EAA are continuously responsible for supporting employers (focus group network partners). The Employment Agencies also advise employers and involve the EAA in the advisory process when employers are to be presented with a “complete package”. From the perspective of the Employment Agencies, the EAA are colleagues with whom “cases are regularly discussed” (focus group network partners).

Specialist Agency representatives described the EAA as a “link” between themselves and the Employment Agencies. Employers typically approach Employment Agencies, who then refer them to Specialist Agencies. These agencies often automatically call in the EAA, which supports employers as a kind of “pathfinder” in the event of possible difficulties, as well as with the application process to Employment Agencies, pension insurance institutions, the Inclusion Office, and the Specialist Agencies themselves (focus group network partners). According to the Specialist Agencies, all network partners work towards the shared goal of promoting sustained employment for people with disabilities:

“Today, we are all working towards the same goal. Our job, regardless of who it is, is to get people into work or keep them in work, and the nice thing about us, at least, is that we all seem to tick in the same direction” (network partner focus group).

Within the Employment Agencies, the Reha SB departments occupy a more central and densely connected position than the Employer Service (AG Service) departments. This may be attributed to staffing differences, as Reha SB departments tend to be better resourced than AG Service departments. Greater staffing capacity likely enables more intensive participation in network activities and broader collaboration with other stakeholders.

Compared to other institutions in the network, the Specialist Agencies and Integration Services are more extensively staffed, providing them with broader reach and the capacity for deeper engagement in network collaboration. Both agencies play a dual role by offering advisory services to employers and to individuals with disabilities. This dual function strengthens their position within the network, as they can thus play a central mediating role between the various groups of stakeholders. Additionally, their function as cost bearers further increases their importance and influence within the ecosystem.

The LVR Inclusion Office was identified as highly important by all of the EAA's most important network partners. This likely reflects the department's central role in processing applications, facilitating access to funding and support services through streamlined, low-threshold application procedures.

To allocate the 18 network partners of the core network (see Figure 2) along the quadruple helix model, the Employment Agencies and the Inclusion Office were counted individually (without differentiation into subdivisions). The EAA itself also belongs to the political sector. Of the *N* = 18 network partners in the EAA's core network (see Figure 2), six network partners are assigned to the politics sector and four (HWK, IHK, LWK and employers’ and entrepreneurs’ associations) to the economy sector. Two actors form the mixed category “Others” and cannot be clearly classified. No actors were assigned to the civil society or academia sectors. The five most important network partners are all assigned to the policy sector.

### 3.3 Cooperation, communication and the exchange of information

1. How is cooperation, communication and the exchange of information between the EAA and its most important network partners in the vocational rehabilitation and participation ecosystem structured?

#### 3.3.1 Cooperation

The working relationship and efficiency of the collaboration between the EAA and the most important network partners was rated on average, from both perspectives, as rather solid and efficient (see Table 3).

**Table 3:** Working relationship and efficiency of the collaboration.

Network partners	EAA			EAA's network partners		
	*N*	*M*	*SD*	*N*	*M*	*SD*
Working relationship						
LVR Inclusion Office	44	3.455	0.663	68	3.515	0.837
Integration Services	17	2.824	0.951	110	3.018	1.04
Specialist Agencies	14	3.357	0.842	84	3.238	0.913
Employment Agencies	24	2.583	1.018	48	3.271	0.676
Efficiency of the collaboration						
LVR Inclusion Office	44	3.909	0.291	68	3.809	0.497
Integration Services	17	3.353	0.702	110	3.155	0.9
Specialist Agencies	14	3.643	0.633	84	3.571	0.749
Employment Agencies	24	2.958	1.042	48	3.458	0.651

*N* refers to the number of ratings given. Response format for frequency of working relationship: 1 = fragile; 4 = stable. Response format for efficiency of the collaboration: 1 = inefficient; 4 = efficient.

From the perspective of the EAA steering group, this result is attributed to many years of collaboration (participatory dialogical process), as the EAA in the Rhineland builds on a predecessor structure established over 20 years ago (5). The importance of the predecessor structure for joint collaborative work is also emphasized in the focus group interviews with the EAA's network partners. According to the Specialist Agencies, there is already a long-standing tradition of cooperation, and the EAA are viewed as “actually..nothing new” (focus group network partners). One network partner elaborated:

“Inclusion advisors at the chambers, that means the Chamber of Crafts and Trades and so on, and these are practically the colleagues who are now also the EAA. So, we already have a relatively long tradition of working together, even if their role was somewhat different before. But in principle, the contacts between us have existed for many years and are well established” (network partner focus group).

The following quote from a network partner exemplifies the quality of the collaboration between the EAA and the most important network partners: “I genuinely find pleasure in collaborating with the EAA”.

#### 3.3.2 Communication

Typically, communication between the EAA and the most important network partners occurs on a monthly basis or less frequently. The frequency of communication is estimated to be higher based on the EAA (see Table 4). The high communication frequency with Integration Services and the LVR is unsurprising, given the EAA's direct institutional links to both. The Integration Services occupy a central position in the EAA network due to their role in the vocational rehabilitation and participation ecosystem. Additionally, Integration Services function as a service provider, employing several EAA advisors. With regard to the form of communication, it can be stated that this tends to be rather formal. This finding is substantiated by the participatory dialogical procedure, which utilizes objects of communication, such as the discussion of applications, to facilitate understanding.

**Table 4:** Frequency and form of communication.

Network partners	EAA			EAA's network partners		
	*N*	*M*	*SD*	*N*	*M*	*SD*
Frequency of communication						
LVR inclusion office	44	2.136	0.824	68	1.338	0.476
Integration services	17	2.529	0.8	97	1.412	0.676
Specialist agencies	14	2	0.555	84	1.267	0.519
Employment agencies	24	1.667	0.868	48	1.354	0.601
Form of communication						
LVR inclusion office	44	2.568	0.998	68	2.706	1.038
Integration services	17	2.765	0.753	110	2.373	1.012
Specialist agencies	14	2.857	1,1	85	2.635	0.924
Employment agencies	24	2.083	1.06	48	2.5	1.072

*N* refers to the number of ratings given. Response format for frequency of communication: daily = 4; weekly = 3; monthly = 2; less frequently = 1. Response format for form of communication: 1 = formal; 4 = informal.

#### 3.3.3 Information processes

The exchange of information was rated as rather quick on average from both perspectives (see Table 5). This result is also reflected in the qualitative interview data. From the perspective of the Specialist Agencies, for example, information is exchanged quickly and via short official channels. These social practices are facilitated by functioning network structures:

**Table 5:** Information exchange.

Network partners	EAA			EAA's network partners		
	*N*	*M*	*SD*	*N*	*M*	*SD*
Speed information exchange						
LVR inclusion office	44	3.546	0.627	68	3.382	0.754
Integration services	17	2.941	0.827	97	3.289	0.735
Specialist agencies	14	3.214	0.802	84	3.452	0.568
Employment agencies	24	2.792	0.833	48	3.375	0.57
Reciprocity of information exchange						
LVR inclusion office	44	3.341	0.987	68	2.618	1.159
Integration services	17	3.412	0.712	95	3.105	0.765
Specialist agencies	14	3.571	0.852	84	2.762	0.83
Employment agencies	24	3.292	1.233	45	3.4	0.58
Exclusivity of the information						
LVR inclusion office	44	3.205	0.632	68	2.441	0.632
Integration services	17	2.118	0.6	95	1.905	0.745
Specialist agencies	14	2.929	0.917	84	2.06	0.665
Employment agencies	24	2.708	0.955	45	2.267	0.618

*N* refers to the number of ratings given. Response format for speed information exchange: 1 = very slow; 4 = very fast Response format for reciprocity of information exchange: 1 = The actor mainly provides me with information; 3 = Our exchange of information is mutual; 5 = I mainly provide the actor with information. Response format for exclusivity of the information: 1 = rather less exclusive; 4 = very exclusive.

network partners). One network partner elaborated:

“We also have these coordination circles and network meetings and so on, where the colleagues also come along..which of course also makes cooperation relatively easy. So, if you need any information or want to coordinate appointments, you just pick up the phone and the matter is actually settled” (network partner focus group).

Furthermore, the provision of information is on average based on reciprocity. Within the participatory dialogic research process, this reciprocal exchange was interpreted as a consequence of the high complexity of the vocational rehabilitation and participation system. Accordingly, the complexity of the system requires that issues and responsibilities be clarified in coordination with multiple institutions.

EAA counselors rated the information they receive from network partners as somewhat more exclusive, on average, than the information they provide in return. The EAA requires information from other stakeholders in the ecosystem for its work. This is in line with the EAA's legal mandate (participatory dialogue procedure). Due to its advisory and support mandate, but above all due to its guiding function, one task of the EAA is to collect and bundle information in order to be able to offer well-founded recommendations on the topic of employment of people with disabilities on this basis.

Integration Services represent an exception in terms of information exclusivity. On average, the EAA rated the information they receive from the Integration Services as less exclusive. Similarly, Integration Services rated, the information they receive from the EAA as relatively non-exclusive. In the participatory dialogue process, it was reconstructed that this result is hardly surprising. The reason given was that there is a large overlap between the EAA and the Integration Services with regard to knowledge about support services and forms of disability. However, the focus group with the EAA counselors revealed discrepancies between the EAA and Integration Services. It was emphasized that Integration Services advisors do not offer the same services as EAA counselors (focus group EAA counselors). A difference between the two institutions lies in their legal legitimacy. The EAA's advisory services are exclusively available to employers and function as independent guides, devoid of any operational influence in relation to the adjudication or refusal of applications. In contrast, Integration Services offers support and advice to affected individuals and also act as cost bearers (York under review). According to the EAA steering group, this is also reflected in their self-image. Although both institutions offer employer counseling, the self-image of the EAA and the Integration Services diverge. The Integration Services primarily represent the position and perspective of those affected, while the EAA act as experts in certain occupational fields and take on and represent the perspective of employers (participatory dialogical procedure). The EAA and the Integration Services represent a complementary unit in their respective perspectives (participatory-dialogical procedure). In the focus group interviews, one EAA counselor reported that it was first clarified that the Integration Services advisors are not obliged to forward all employer cases to the EAA, but rather that a targeted contact should be established: “Of course the cases will continue to go to them, and if I am specifically asked for, it would be nice if I am informed or included, that we also cooperate in cases” (focus group EAA counselors).

In comparison, the findings indicate that the contact between the Employment Agencies and the EAA is initiated by the Employment Agencies. EAA counselors stated that the Employment Agencies refer those affected to the EAA, as the EAA have knowledge and contacts that the Employment Agencies employees do not have (focus group EAA counselors). In addition, there are requests from the Employment Agencies for company contacts: “For example, ideas on where else you could apply or something, because such requests come to me, sometimes also from the Employment Agency, from the rehab advisors, whether I have any ideas on which companies you could still contact” (focus group EAA counselors). Information is also requested that is not explicitly stated in the legal mandate. One EAA counselor reported that intensive contact with the Employment Agencies had been initiated in some regions and that this contact now “seems to be bearing fruit”, as more inquiries are coming from the employment agency (focus group EAA counselors). The perspective of the EAA is also confirmed by the assessment of the Employment Agencies. Based on the Employment Agencies, the EAA are perceived as colleagues with whom a regular exchange takes place. In addition, the EAA would be directly involved in the context of employer consultation if a “complete package” (focus group network partners) is to be presented to employers:

“Well, for me personally, that’s simply part of the complete consultation. As I just said, I see myself as having an active duty to provide advice and my employer doesn't know its way around this jungle, and that’s our job, to say we can help you in this and that area, and you also have other partners here” (focus group network partners).

The exchange of information between the EAA and the Specialist Agencies is also described as reciprocal. There is a slight tendency here towards the EAA: The EAA provide the Specialist Agencies with slightly more information than the Specialist Agencies provides the EAA. At the same time, the information that the EAA receives from the Specialist Agencies is considered to be more exclusive than the other way around. In this regard, the Specialist Agencies appear to demonstrate a reduced need for information from the EAA. One reason for this pattern could lie in the function and tasks of the EAA. When asked about the cooperation between the Specialist Agencies and the EAA, the interviews describe the EAA (from the perspective of the Specialist Agencies) as acting as a “link” between the Employment Agency and the Specialist Agencies. The fact that certain applications are also submitted to the Specialist Agencies could explain the relationship between the Specialist Agencies and the EAA, as well as the different functions in the exchange of information. The EAA provides the Specialist Agencies with relevant information when applications are submitted, and the EAA's familiarity with the vocational rehabilitation and participation system ensures the applications are completed accurately, thereby reducing the need for further clarification on the part of the Specialist Agencies (focus group network partners). For example, the necessary documents are collected by the EAA without them having to check with the Specialist Agencies (focus group network partners).

## 4 Discussion

### 4.1 Innovation through networking

From an innovation theory perspective, the current networking of the EAA is relevant, as is its future potential to develop the vocational rehabilitation and participation ecosystem through targeted networking. Analyses to date show that the EAA are most closely networked with political stakeholders, including Integration Services, departments of the LVR Inclusion Office, Specialist Agencies, and Employment Agencies (31). This indicates that the EAA are primarily connected to stakeholders whose services they are legally mandated to recommend to employers [Section 185a (2) SGB IX]. This strong connection to the policy helix sector is understandable, but also poses a challenge, because the vocational rehabilitation and participation ecosystem is already equipped with established stakeholders with a legal mandate to advise employers (5). However, implementing the EAA has made the vocational rehabilitation and participation system even more complex, despite its pilot function being intended to improve navigation within this complexity (5). Organizational theory describes this dual effect as a form of ambiguity: on one hand, the EAA expand the system; on the other, they enhance orientation within it. Through their pilot function, they help simplify processes for employers, improve procedures, and facilitate navigation through the complex landscape.

However, the EAA's legal mandate is also to approach employers and raise their awareness of the training, recruitment and employment of severely disabled people (Section 185a SGB IX). In the helix model, employers are considered part of the economy. Closer networking with their interest groups—such as employers and business associations—could therefore be particularly worthwhile. However, employers’ and business associations only occupy a peripheral position in the EAA's current networks. The employers’ and business associations themselves did not take part in the survey and were only rated as important by eight stakeholders within the network. From the perspective of the network partners in the vocational rehabilitation and participation system, employers’ and business associations therefore do not appear to be among the central network partners. This is surprising insofar as employers are the key stakeholders when it comes to real participation in work. From the perspective of the EAA, only one person in the online survey rated the employer or business association as “very important” for their own work. The fact that the employers’ and business associations themselves did not take part in the survey is most likely due to the fact that they were not invited to participate by the EAA consultants. This is because the EAA only forwarded the invitation to the survey to their very important network partners. From an innovation theory perspective, it could make sense to involve the employers’ and business associations more closely in the network and thus establish network partnerships in the economy. This approach would also be in line with the preferences of employers, who stated in the interview that it would be attractive to be proactively addressed by the EAA and informed about employment opportunities for people with disabilities: “But as an employer [..] you are not actively addressed [..]. So perhaps more could be done, for example approaching employers [..] and the topic is simply not present. And I think more could be done about that. So maybe, it's tedious to write to every employer, I can see that, but maybe there are events, like the Trade Fair or something. Employer career days, I don't know, stock exchanges or something like that” (Interview with employer).

Civil society stakeholders are not yet represented in the EAA's core network. Although not explicitly stated in the EAA's legal mandate, it seems sensible from an innovation theory perspective to actively involve civil society stakeholders, such as self-advocacy organizations or associations of people with disabilities, in the EAA network. A current component of the EAA's core network is already the Supplementary Independent Participation Counseling (EUTB), which was implemented in 2018 via § 31 SGB IX as a counseling service for people with disabilities by people with disabilities (peer counseling) in the (vocational) rehabilitation and participation system (40). According to the prevailing legal framework, EUTB are allocated to the policy sector. These entities are obligated to provide benefits exclusively to the designated beneficiaries. They offer assistance through various forms of support, including planning, orientation, and decision-making assistance, even prior to the submission of applications for specific benefits for participation (41). The EUTB could act as multipliers for the helix sector civil society—similar to the employers’ and business associations for the politics sector.

Stakeholders from the academic sector are currently not part of the EAA's core network. However, from an innovation theory perspective, incorporating expertise from this sector into the EAA implementation process appears both reasonable and beneficial. This is already underway in the Rhineland region in North Rhine-Westphalia through a formative evaluation of the EAA's implementation. Scientific studies—in particular on the impact of the EAA on the participation of people with disabilities in working life—should be initiated at the national level. Furthermore, establishing connections with institutions offering vocational education and training, such as schools, vocational colleges, and further education providers, would facilitate the communication of practical concerns and employer needs regarding the qualification of future trainees or employees in the academic sector.

### 4.2 Limitations

The EAA network structures in the Rhineland were surveyed in 2023. On one hand, the EAA is a recently implemented labor market policy instrument (2022); on the other, networks are inherently dynamic in nature (38). This also applies to the EAA in the Rhineland, as confirmed by EAA counselors. It can therefore be expected that additional structures may emerge in future survey phases. To address this limitation and track changes over time, the egocentric networks will be resurveyed in 2025 and analyzed for differences and developments. For this purpose, the already tested expert workshop (30) from the year 2023 will be conducted again with the EAA counselors with a new focus in terms of content.

In addition, the size of the institutions in the core network varies greatly. This applies both to the number of institutions and to the departments within the institutions. For example, the Specialist Agencies and Integration Services are significantly larger institutions than, for example, the Action Inclusion by the Inclusion Office. The number of institutions also varies depending on the region. These different structural conditions must be taken into account when classifying and evaluating the positioning of the stakeholders within the network.

Another limitation is the small sample size, which potentially impairs the generalizability of the results. At the time of the survey, 20 counselors were working for the EAA in the Rhineland, with a response rate of 90%. To replicate the results (also for other locations), studies must be conducted with additional samples.

There is also a risk of selection effects, as the selection of participants or cases may not have been completely random or representative, which could potentially lead to distortions in the results. For data protection reasons, it was not possible to clearly match the data between the EAA specialist advisors and their network partners. These limitations should be addressed in future studies in order to enable a more robust and comprehensive analysis.

The results of the qualitative content analysis must also be viewed against the background of a number of limitations. The combination of deductive and inductive category formation invariably entails the potential for unexpected topics to be addressed only to a limited extent. In addition, the results can only be generalized to a limited extent, as they are context-dependent on the one hand and shaped by individual perception on the other. Furthermore, the interviewees were drawn at random, meaning that not all people in the surveyed groups—for example, not all EAA consultants or all network partners—were interviewed. The random selection of interviewees may have resulted in people being interviewed who, for example, are particularly well networked. In order to validate the results, networks of other people will be surveyed and analyzed in the future.

### 4.3 Practical implications and outlook

Practical questions emerge regarding how the EAA can build a network with stakeholders from helix sectors that are currently underrepresented or absent from the core network. The potential value of networking the EAA with employer and business associations has already been argued. When network maps were collected, only six EAA counselors rated employer and business associations as relevant to their work. Notably, with one exception, these counselors were employed by employer-related institutions such as the Chamber of Skilled Crafts [Handwerkskammer (HWK)], the Chamber of Industry and Commerce [Industrie- und Handelskammer (IHK)], or the Advanced Training Academy for Business [Fortbildungsakademie der Wirtschaft (FAW)]. Therefore, the counselors at this institution could act as “door openers”, thereby facilitating stronger networking between the EAA and employers’ or business associations. This strategy could help strengthen the position of employer and business associations within the network. In the future, additional strategies for engaging employers—such as presenting the EAA's services at established events and employer forums—should be explored by EAA counselors.

Connections with civil society actors—particularly individuals with disabilities—could also be initiated through stronger collaboration with the EUTB. Taking into account the perspectives of people with disabilities on the subject of participation in employment, for example through participatory procedures and processes, can help to establish advisory processes that address the needs of people with disabilities more precisely.

It is not yet clear whether the EAA, with its strategy of networking with key stakeholders in the complex vocational rehabilitation and participation ecosystem, will be able to make a significant contribution to increasing participation opportunities for people with severe disabilities. Subsequent research projects should examine whether and how employers are sensitized to the training, recruitment, and employment of severely disabled people, as well as the process evaluations of the still-young innovation EAA (42).

## Data Availability

The original contributions presented in the study are included in the article/Supplementary Material, further inquiries can be directed to the corresponding author.

## Footnotes

1 This article mostly uses the term “people with disabilities” as it is used in the Federal Participation Act and the UN Convention on the Rights of Persons with Disabilities and as demanded by self-advocacy organizations. The term “severely disabled persons” is always used when referring to official statistics or legal texts that use the construct “severe disability”.

2 York J, Sartor T, Lamb S, Schulze S, Jochmaring J, Kuhn JT, et al. Innovation Biography as an Instrument to Evaluate Inclusion Policy: The Case of Single Contact Points for Employers, Germany.
